# Preparation of the Mouth for the Insertion of Artificial Teeth

**Published:** 1884-12

**Authors:** W. H. Atkinson

**Affiliations:** New York.


ARTICLE II.

### PREPARATION OF THE MOUTH FOR THE INSERTION OF TEETH OF SUBSTITUTION.

BY W. H. ATKINSON, M. D., D. D. S., NEW YORK.

The first requisition is to ascertain the impracticability of the preservation of the neutral teeth.

When it is decided that one or more teeth in the mouth are beyond natural repair, it becomes necessary to ascertain the extent of the defect—that is to say whether the crown and root are so far deteriorated as to render it impossible to tolerate them, one or both.

In case the crown alone has to be given up, and the root capable of being rendered compatible, then it should be so prepared as to give the best support possible to an artificial crown.

In case the pulp be living, we have the opportunity to supply a really first-class substitute by the use of a banded crown. The mode of preparation of the root in this case consists in so trimming the neck of the root and stub of the crown as to afford as nearly as possible a cylindrical form of base upon which to adjust a perfectly fitting band so as firmly to retain the crown.

In case the pulp be dried, a very different procedure is demanded. And we now may resort to band alone as before, or band and pivot or screw into the canal by which to retain the substitute firmly in place.

In case we resort to band without pivot or screw, it will be necessary to so treat and fill the canal as to preclude the advent of subsequent alveolar abscess. This preparation will consist in taking out every vestige of dead and foreign substance from the entire length of the canal, and after disinfecting completely, hermetically sealing it, then proceed as in the first instance.

When it is deemed best to use the pivot or screw alone without any band, the root should be dealt with as before, with the exception of only filling the end of the root and so much as is intended to be above the pivot or screw. And then the base of the root should be so hollowed out as to afford a pocket around the pivot hole of such dimensions as to secure a good hold between crown and root by some tough and indestructible cement—gutta percha, oxychloride or oxy-phosphate of zinc or amalgam, as the operator selects.

Where both band and pivot are resorted to, the procedure is so nearly the same as just detailed as to not demand repeating.

If band and screw be preferred, the difference of manipulation will consist in following former directions in putting on the banded crown with cement and afterward sending the screw firmly into the already prepared hole in the root, being careful to cut it long enough to allow it to come fully through the cement within the band, thus affording a good finish. The cement may be finished off even with the end of the screw, or may be cut away, and gold, tin or amalgam used to fill in the band around the screw up to its end or over it, as preferred, and thus secure a complete restoration of the natural contour.

When roots are to be removed an impression should be taken and substitutes prepared before the extraction, so as to insert the substitutes immediately upon cessation of hemorrhage after extraction.

This is regdared as the only humane and artistic method, as it controls the extent of absorption and prevents the falling in of the lips, as invariably follows the old mode of removal and waiting for the healing of the gums and removal of the sockets.

The case where all the teeth and roots are so far gone as to demand removal are so rare as to render it unlikely that a well educated dentist at this day will be found to advise it.

But even in such rare event the above commended methods of preparing the mouth for artificial substitutes still hold good.

The preparation of roots for sustaining partial or entire sets of substitutes differs in method according to the movable or immovable character of the pieces.

When they are not intended to be taken out for cleansing they will be permanently attached to the roots as before described.

Where they are to be taken out it will be necessary to securely insert sockets (of gold or platinum) in the roots into which to pass the pivots which have been soldered to the pieces, complete or partial, so as to hold them firmly in place.

These pivots may be solid wires or cylinders, and accurately adjusted to the cylinder sockets in the roots; or they may be slit at the ends and sprung apart so as to be retained by the tension of the spring. The extreme points must be beveled to admit them when pressed together at the time of insertion.

The importance of the preservation of the roots in their sockets has not been adequately appreciated in the past history of artificial dentistry, even among the more learned and skillful in the practice.

The principal duty of the roots of the teeth is the reception and distribution of the force of the jaws in mastication, which is never even approached in its perfection by substitutes, which rest upon the gums as is inevitable where all the roots are gone.

To understand the changes which occur upon the removal of the roots of the teeth it is necessary to know the changes which take place in their development, nutrition and the absorption of the now no longer needed sockets in which they were held.

This knowledge involves study of food, blood, tissue and molecular metamorphoses by which it is effected.

The main obstacle in the way of study and teaching the changes of functioning bodies and processes, consists in the methods and classifications resorted to by those who are most earnest and persistent in the research. Individual and separate observation tends to one-sided and incomplete interpretation of fact and function, while investigations made in classes of compatible associates tend to breadth and clarity of view and interpretation of the facts and phenomena observed.

A perfect peptone is a completely eleborated pabulum capable of absorption by needy tissues without awakening any septic or retrogressive action in the territory where it may be introduced through the natural channels or by artificial injection.

The mollusks used for food are so nearly reduced to peptones that they demand but little expense of digestory energy to fit them for assimilation by hungry tissue.

A fresh raw oyster is a good example of a nearly perfect peptone easily disposed of by even weak digestory apparatus. Hunter says "there is not a natural action in the body, whether voluntary or involuntary, that may not be influenced by the peculiar state of the mind at the time." This is evidence of the illumination under which he wrote, of which he was aware but unable to refer to its source, in his inspiration of the radiance impact which revealed it to his waiting consciousness.

He was a man of such crotchety wilfulness that he implicitly followed his illumination and believed in it as his own genius without caring to stop to consider. This grew out of his solitary habit of investigation and unwillingness to admit others' observations as valid.

The domination of authority, then more than now, may be cried as extenuation of his absolutism, which had produced the "state of the mind at the time" to which he referred, and of which he seemed unconscious in his personal experience, though so clearly perceiving it in others.

Food must be digested into blood, blood into pabulum, pabulum into protoplasm, protoplasm into embyronal corpuscles and these into tissues.

To show all this in a short paper to be read and discussed at a meeting where many other subjects are to be heard, is quite out of the range of possibility, even by one capable of doing such an herculean work.

But let us at least take a few steps in the direction of attempting this transcendently important foundation work. We may not live to see the building complete, yet we may lay some of the foundation stones upon which the magnificent temple is to stand as a beacon set upon a hill, whose light will disperse the darkness that has hitherto made empiricism rather than science a guide in practice.

Digestory and blood changes consist of metamorphoses in food, pabulum and protoplasm.

Let us begin with chyme, which is divisible into four distinct proximate principles: 1. Dis-peptote (division of chyme by splitting up of its molecules.) 2. Para-peptone (combining by sidewise laying of molecules.) 3. Meta-peptone (closer combination by getting between the molecules,) and 4. Perfect peptone (in which the molecules get into such intimate relations as to admit of absorption by the needy tissues or passage into chyle, the absorption of which in the circulation keeps up the blood supply.)

The changes which accompany absorption and intimate admixture of chyle with venous blood are not yet easily provable as separate and conjoined steps or stages of awaking and engagement of bonds of afinities, molecular and atomic, as they have been in those occurring in chyme producing the varieties of peptones.

When chyle is well diffused in the venous blood by running its course from the receptaculum chyli to the sub-clavian, vena cava and heart, it is ready to be sent to the lungs, there to be brought within the sphere of influence of the vivifying oxygen, to be acted upon and to act by its presence in the capillaries in the walls of the air vesicles where two degrees of blending of oxygen and blood take place.

One may be called oxygenation, the other oxidation ; the first being a sort of sidewise laying of the molecules of oxygen about the red blood corpuscles, the other a completion of combustion of the partly burnt carbon in the venous blood.

This imperfect conversion of carbon lays the foundation for all inception of blood sepsis. The word vivification has not been sufficiently defined and limited to enable the beginner to utilize it in the legitimate sphere of its activity. Vivification is the making alive the bodies (called molecule, granule, corpuscle, tissue, organ and consciousness) in which functional activity takes place.

To comprehend the degrees of combinations and separations of the elements of functionary bodies, it is necessary to have a point of beginning for the movements, the modes and measures of which construct and operate these factors of function. This beginning point has been accepted and named atom. This is the unit, aggregations of which present us with material bodies.

The capability of being combined and separated is in the atom.

The *form* of combination taken on depends upon influx, the mode and degree of which constructs and limits the type or form of the body and the measure or sort of function it can operate.

Influx is radiations coming from the sun (where the sun gets it is a disputed point.)

We may say Solar Radiance operates upon atomic mass, producing molecular mass, of which sensible or perceptible bodies are constructed. The first is an aggregation of molecules arresting the light so as to cast a shadow, which reveals the *granules*, &c. Construction and destruction then, of planetary bodies consists in putting on and taking off tensions of radiancy. The *embodiment* of these tensions constitutes consciousness.

So we see that "The All" is infinitesimally embodied in the least as well as in the largest functioning bodies. Furthermore, we have not yet arrived at actually accurate and lucid limitations and definitions of the finer steps of career. And we are often lead to nominate as primal or first, bodies and processes that are but sequences of indefinite lines of ancestral or causative antecedents. The individualities cognizable to us are not single and unitary, but very multiple and complicated in structure and character of bodily presence and activity. Even the body agreed to be put for first in the series, is by no means unitary in character, but at least binary if not ternary. It has a donamic and static aspect, and some say a conscious aspect of personal presence. Whatever cannot be said of a primate in character and principle cannot be predicated of its aggregates.

Sepsis is evidence of imperfect digestory eleboration of chyme, peptones, chyle, blood and pabulum, as it is a retrogressive mode and irregular manner of molecular metamorphosis in the preparing of massfood for the use of the elements of the body in which the functional changes occur, known as molecular, granular, corpuscular, organic, systemic and conscious changes.

Digestion is a sort of churning, shaking together of the food until it becomes, in some sort, a series of soups that have been named variously by different investigators, viz: Chyme (divisible into dys, para, meta, and perfect peptones,)

chyle and blood, which, by a retrogressive movement or back lash in molecular agitations becomes converted into pabulum, protoplasm and indifferent, or embryonal corpuscles, sometimes called mucous globules and animal-cells. These hold the values of the impacts of the energy which operates—breathing as the first example of impact of energy, the degrees and modes which constitute the initial of every change, molecular or massive, each body above acting as sun to that below it and each below acting as planet to that above it.

The accumulations and storing of this (to functional bodies) primal source of power, constitutes the measures of personal presence, potential and active, mental and corporeal, each involving the other in alternations of predominant and subdominant necessity!

When the radiancy (taken in and stored by breathing) is needed to perform any act of functioning body, the need awakens the tension of desire between the stored radiancy and the debilitated body, and thus the solar fullness flows into the planetary voidness satisfying the demand.

Where the channels of respiration are reduced in activity or in caliber, so as to minify the supply of air to the part, the stored energy takes the place of that which should have been received, thus supplying the functional power in the active tissues. This induces debility to the degree of disturbance of functional rythm and is manifested in spasmodic efforts to attain full supply of air or to eject the obstructions in the channels by retching, coughing, sneezing, &c.

This may occur in the minutest or in the most massive examples of respiratory, circulatory, innervatory, digestory or nutritive channels, disturbing the diamagnetic polarizations upon which functional changes depend in molecular, corpuscular, tissual, organic, systemic and conscious degrees.

Whenever these reductions of power or capacity (caliber) occur below the conscious degree they are said to be automatic and spontaneous and to control functional actions until they invade the plane of consciousness, where they may be recognized and in turn controlled.

The consciousness is awakened in the emotional, ideational, consenual, motor, excitor and voluntary degrees of automatism that turns the attention of the consciousness to the stored knowledge of the functions and factors of function in coinciding and opposing degrees, thus appearing as will, which has two modes. The first mode of stored knowledge is spontaneous or unconscious-consciousness, and involuntary, or sub-will. The second is the act of awakened purpose, or will proper.

I say "will proper" in defcrence to extant classifications which ignore the minor manifestations of *purposive energy* in the construction of molecules, granules, corpuscles, tissues and organs in a system in which it is said the consciousness *per se* has its seat, from which it sends forth behests in the form of will (volition.) In the massive sense this is true. Works on physics and science limit all demonstrations to the crudity of differential sensuous perception and denounce as "*un*-scientific" all attempts to deal with the primal realm of consciousness, which they use in their ratiocinative processes and instantly ignore.

*Will* is the RADIANT Autocrat in every realm wherever function of any sort is enacted, be it in pure consciousness or in any of its differential expressions. The great difficulty of comprehending and teaching Physiology, a Pathology and Therapy lies right here. So long as we depend upon imperfect classifications of body and process, just so long will we be beating about in a succession of failures to arrive at certainty of origin, career and destiny of function and factor of function. This is proved to be yet our condition respecting *causation* of functional activities and the modifications of *disease*.

The two queries now agitating the minds of enquirers are, first, Are Micro-organisms the primal factors of function? Second, (or) Are they only factors in aberrant functional activity?

When sepsis occurs in molecular mass does it depend on impact from without its own territory, or is it an internal disturbance reducing some molecules to the atomic state? In either case it would be the result of impact from without, the only difference consisting in the openness and the occultness of the altered movement among the constituent elements of the mass.

The internal ability to respond being the conservation or storing of the energy of former impacts, by which the molecular-mass was produced. Ripeness of molecules fits them to respond to the demand of type in the granules which is the first example of visible functioning body. In like manner of the ripening process, granules take on the type of corpuscles and corpuscles that of tissues, and tissues become wrought into organs and these collated into systems of organs, all operated by influx of typal energy by which RADIANCE holds dominion of the departments of functioning bodies.

The ripeness requisite for normal functional change in nutrition and that resulting in rotteness, disintegration, are both dependent upon degrees of satisfaction and dissatisfaction of bonds of combining affinities.

1. Satisfaction of tension is attained when the ripening process moves regularly in disruption and reconstruction of the molecules at the point of nascent condition whereby nutrition is perfected.

2. Dissatisfaction results upon minification of the influx of radiancy to the degree of failing to hold the elements of the tissues in due order of arrangement of corpuscular and granular tensions.

In the main micro-organisms have been regarded as iconoclastic in function, *i. e.*, molecule breakers rather than molecule constructors. Hence the practice of classing them as pathological—disease producing bodies. Those who most strenuously claim that all disease is a result of micro-organisms fail to clearly discriminate and define what are to be catalogued as micro-organisms, capable of interfering with normal nutrient currents.

Micro-cocci are none other than granules, *i. e.*, a mass of molecules dense enough to arrest the light pencil sufficiently to cast a shadow, under a high magnifying power of lenses, whereby it becomes cognizable to the sense of sight. From this initiative of observable activities we may progress with a tolerable degree of certainty as to the characteristics of corpuscles, tissues and their aggregations.

By rigidly keeping in mind the order of formation and metamorphoses in functioning bodies already noted we will be able to appreciate the necessity for room in space and open channels or breathing and feeding to enable type to accomplish its purposes in formation, nutrition, growth and activities of body and mind. If we desire to master the subject of decay, falling apart, we must first comprehend the construction—come-together—of the integers of the bodies we study. To do this in anything like a fullness of detail would far exceed the limits of a single paper. Permit then the attempt at a succinct summation of the integers of body and process in massive and minute manifestation.

Impact of light through ether becomes differentiated into four trinities of expression as follows:

1. Electrism,

 Magnetism,

 Chemism, heat.

2. Breath,

 Blood,

 Body.

3. Respiration,

 Circulation,

 Nutrition.

4. Feeding,

 Innervation,

 Mentation.

Embodiment of these twelve modes of light give their con-cens-ous in individual C. O. N. S. C. I. O. U. S. N. E.

S. S. I, Me. The ego is the sum of minor egos through which all modes are separately and conjointly displayed in manner and degree of bodily and functional presence.

Each individual of simplest or most complex form has sufficient space in which it is a plenum, of its kind, by which it has freedom to act out its natural desire to catch, hold and release the object desired by which it is formed, fed and worded in single, double, triple or multitude lines of tension, intension, contension and extension of plan and purpose of desire constituting career.

Prof. Summers says, "Functional activity is differentiation of integral elements." And Prof. Pepper says that "it is a remarkable fact that when an acid and alkaline solution are so placed that their union may be effected through the substance of an animal membrane, or, indeed, any porous diaphragm, a current of electricity is envolved."

Now, with the exception of the stomach and cæcum, the whole extent of the mucous membrane is, in the human subject, bathed in an alkaline mucous fluid and the external covering of the body, the skin, is as constantly exhaling an acid fluid. The mass of the animal frame is thus placed between the two great envelopes, the one alkaline and the other acid. meeting at the external outlets. This arrangement has been shown by Donne to be quite competent to the evolution of electricity. The purity and cleanliness then of these two modifiers of molecular combinations and separations, by which the diverse sources of organizing impact are brought into working relations, will give the measure and character of the work effected in them.

As these envelopes, skin and mucous membrane are the products of an arrangement of different corpuscles, the study of this arrangement is the field in which to look for the intiative of perceptible function. And as these final outcomes of the ept-blast and hypo-blast constitute an electromagnetic battery, are we not nearing the seat of nutrient and de-nutrient changes? Infection, contagion, &c., &c.

